# Supplementary material for: Development of Stable Amino-Pyrimidine–Curcumin Analogs: Synthesis, Equilibria in Solution, and Potential Anti-Proliferative Activity
Source: Int J Mol Sci. 2023 Sep 11;24(18):13963. doi: 10.3390/ijms241813963 (PMC10531168; doi:10.3390/ijms241813963)
Supplement: Supplementary file 1 [file ijms-24-13963-s001.zip › ijms-2573884-SI.pdf]

SUPPLEMENTARY MATERIAL

Development of stable amino-pyrimidine curcumin analogs: synthesis, equilibria in solution and preliminary *in vitro* assays.

Matteo Mari[1], Matteo Boniburini[1], Marianna Tosato[1,2], Luca Rigamonti[1], Laura Cuoghi[3], Silvia Belluti[3], Carol Imbriano[3], Giulia Avino[1], Mattia Asti[2], Erika Ferrari[1,*]

[1] Department of Chemical and Geological Sciences-University of Modena and Reggio Emilia, via Campi, 103, 41125, Modena, Italy; erika.ferrari@unimore.it

[2] Radiopharmaceutical Chemistry Section, Nuclear Medicine Unit, Azienda USL-IRCCS Reggio Emilia, via Amendola 2, 42122, Reggio Emilia, Italy; mattia.asti@ausl.re.it

[3] Department of Life Sciences - University of Modena and Reggio Emilia, via Campi, 203, 41125, Modena, Italy; silvia.belluti@unimore.it & carol.imbriano@unimore.it

*Correspondence: erika.ferrari@unimore.it; Tel.: +39 059 2058631

Figure S1. ^{1}H- NMR spectrum of PY1 in DMSO-d_6 at 600 MHz (298K). Highlighting boxes show the resonances of olefinic protons in the *E* configuration.

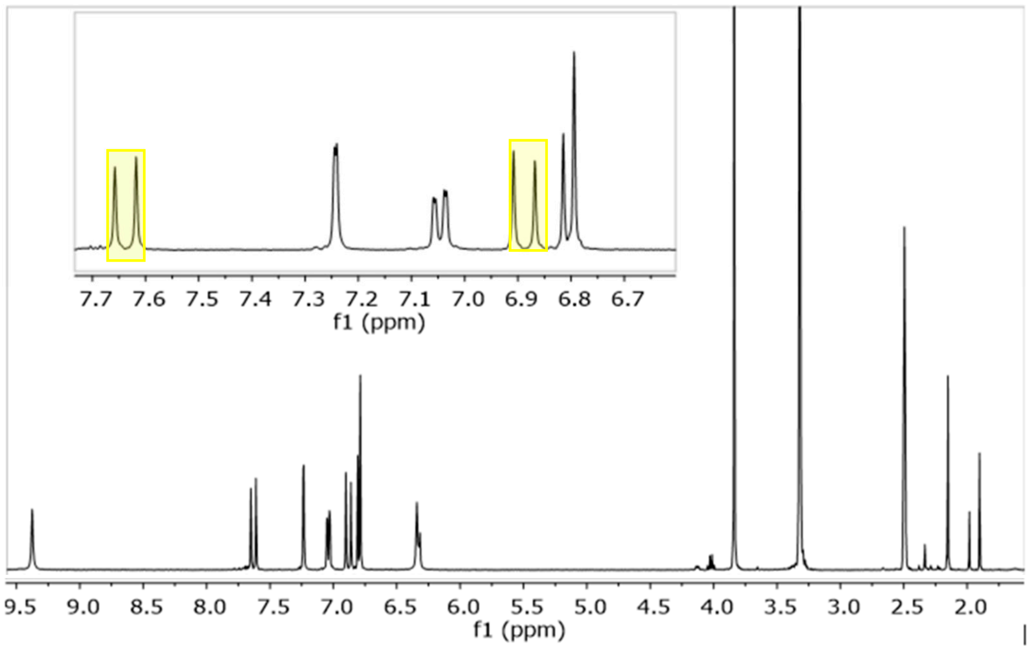

Figure S2. pH-metric spectrophotometric titration of MPY3 at 25°C in aqueous solution ([MPY3] = 50 µM; [$NaNO_3$] = 1 mM), in the 250–650 nm spectral range. Red spectrum pH = 2, green spectrum pH = 7, blue spectrum pH = 11. The inset shows the absorbance *vs*. pH at 355 nm (black) and 406 nm (red). The inset reports the plot of absorbance at 355 and 400 nm *vs.* pH.

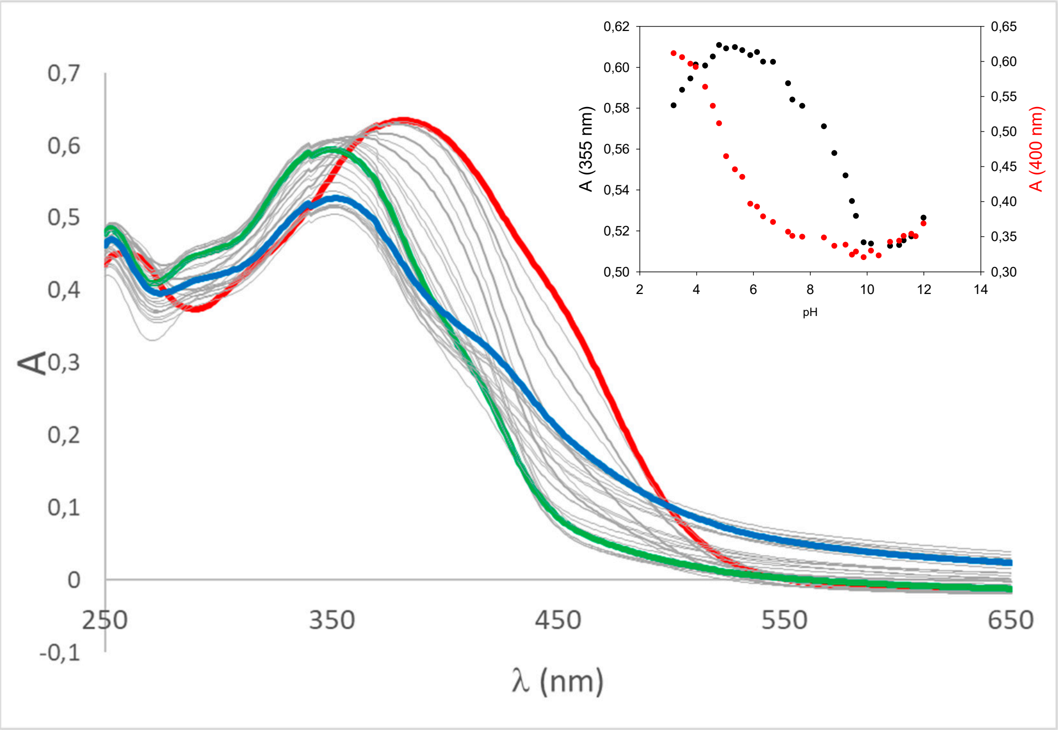

Figure S3. pH-metric spectrophotometric titration of PY1 at 25°C in aqueous solution ([PY1] = 20 µM; [$NaNO_3$] = 1 mM), in the 250–650 nm spectral range. Red spectrum pH = 2, green spectrum pH = 7, blue spectrum pH = 11. The inset shows the absorbance *vs.* pH at 355 nm (black) and 406 nm (red). The inset reports the plot of absorbance at 410 nm *vs.* pH.

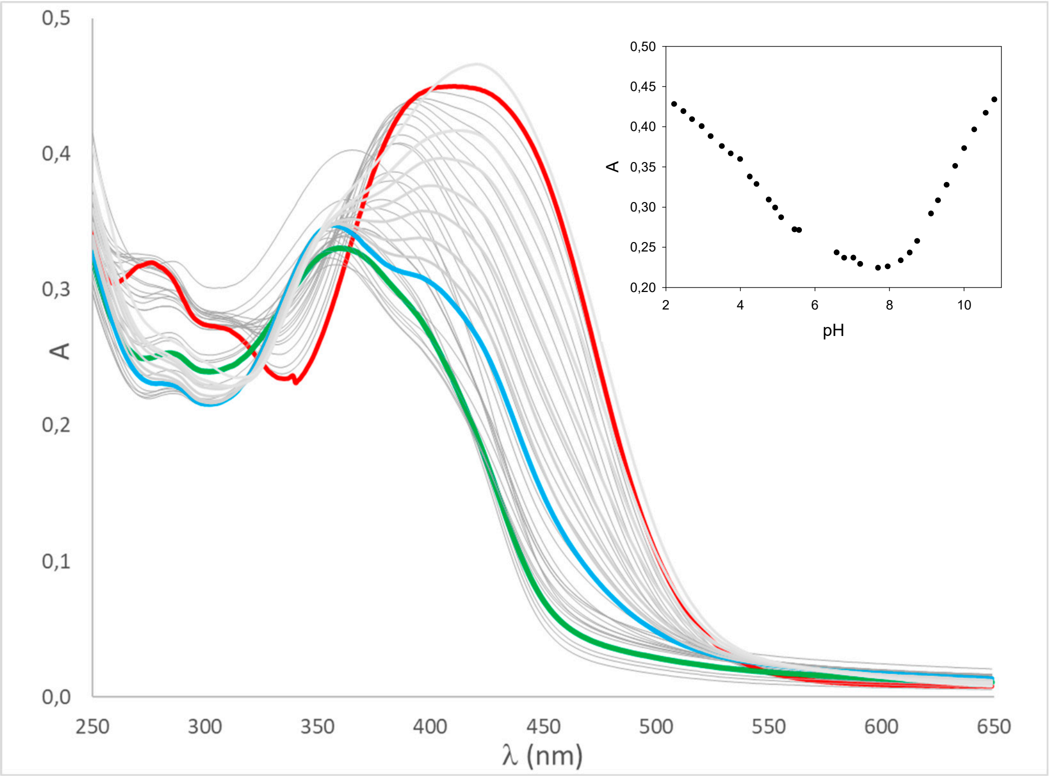

Figure S4: Representative histograms of cell cycle distribution of A) LNCaP, B) PC3 and C) HCT116 cells in control (DMSO) condition and after treatment for 48h with Curcumin or PY3. The different colors represent the percentage of cells in G0/G1 (brown), S (green), G2/M (blue) and SubG1 (pink). D) Table summary of cell cycle distribution in representative experiments (A, B, C).

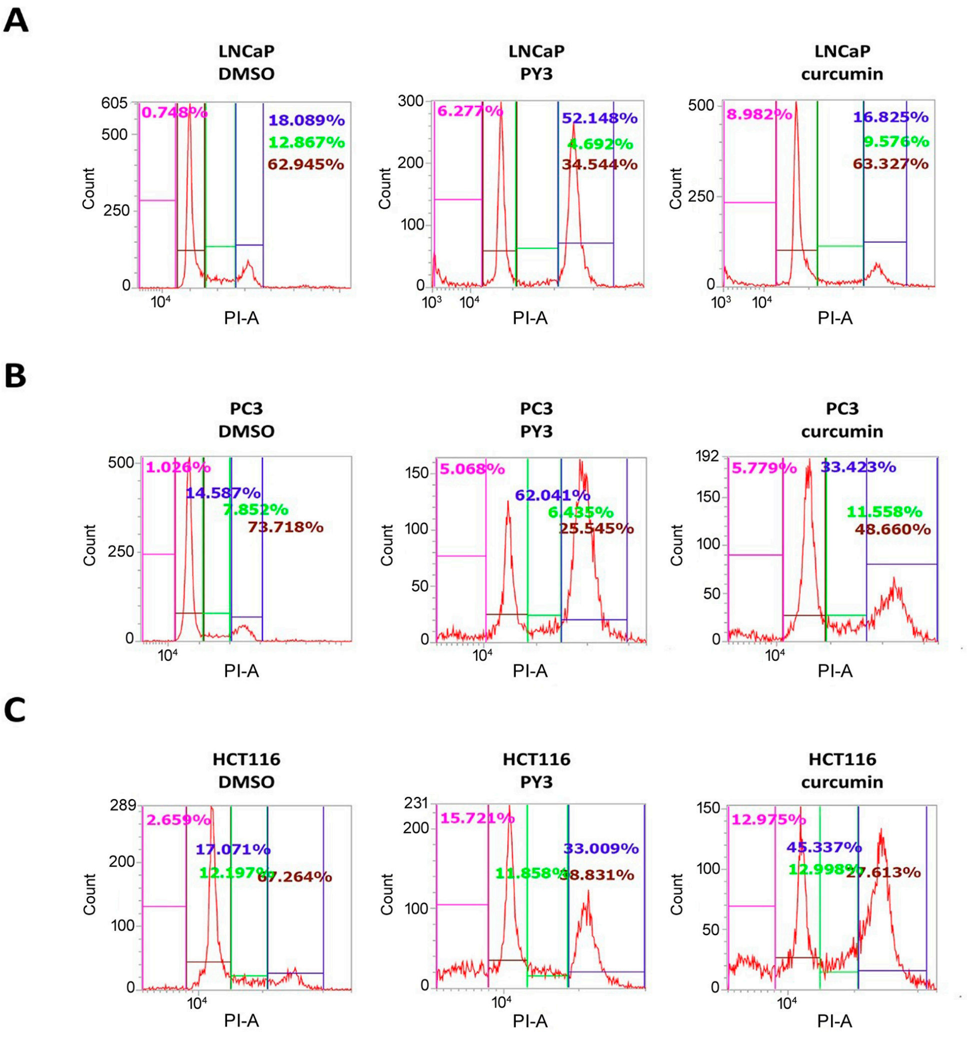

D

Cell Line	Treatment	G0/G1	S	G2/M	SubG1
LNCaP	DMSO	62.95%	12.87%	18.09%	0.75%
	PY3	34.54%	4.69%	52.15%	6.28%
	Curcumin	63.33%	9.58%	16.83%	8.98%
PC3	DMSO	73.72%	7.85%	14.59%	1.03%
	PY3	25.55%	6.44%	62.04%	5.07%
	Curcumin	48.66%	11.56%	33.42%	5.78%
HCT116	DMSO	67.26%	12.20%	17.07%	2.66%
	PY3	38.83%	11.86%	33.01%	15.72%
	Curcumin	27.61%	13.00%	45.34%	12.98%
